# Automated cell-type classification combining dilated convolutional neural networks with label-free acoustic sensing

**DOI:** 10.1038/s41598-022-22075-6

**Published:** 2022-11-18

**Authors:** Hyeon-Ju Jeon, Hae Gyun Lim, K. Kirk Shung, O-Joun Lee, Min Gon Kim

**Affiliations:** 1 Data Assimilation Group, Korea Institute of Atmospheric Prediction Systems, Seoul, 07071 Republic of Korea; 2 Department of Biomedical Engineering, Pukyong National University, Busan, 48513 Republic of Korea; 3 Department of Biomedical Engineering, University of Southern California, Los Angeles, CA 90089 USA; 4 Department of Artificial Intelligence, The Catholic University of Korea, Bucheon, 14662 Republic of Korea

**Keywords:** Computer science, Biomedical engineering, Electrical and electronic engineering

## Abstract

This study aimed to automatically classify live cells based on their cell type by analyzing the patterns of backscattered signals of cells with minimal effect on normal cell physiology and activity. Our previous studies have demonstrated that label-free acoustic sensing using high-frequency ultrasound at a high pulse repetition frequency (PRF) can capture and analyze a single object from a heterogeneous sample. However, eliminating possible errors in the manual setting and time-consuming processes when postprocessing integrated backscattering (IB) coefficients of backscattered signals is crucial. In this study, an automated cell-type classification system that combines a label-free acoustic sensing technique with deep learning-empowered artificial intelligence models is proposed. We applied an one-dimensional (1D) convolutional autoencoder to denoise the signals and conducted data augmentation based on Gaussian noise injection to enhance the robustness of the proposed classification system to noise. Subsequently, denoised backscattered signals were classified into specific cell types using convolutional neural network (CNN) models for three types of signal data representations, including 1D CNN models for waveform and frequency spectrum analysis and two-dimensional (2D) CNN models for spectrogram analysis. We evaluated the proposed system by classifying two types of cells (e.g., RBC and PNT1A) and two types of polystyrene microspheres by analyzing their backscattered signal patterns. We attempted to discover cell physical properties reflected on backscattered signals by controlling experimental variables, such as diameter and structure material. We further evaluated the effectiveness of the neural network models and efficacy of data representations by comparing their accuracy with that of baseline methods. Therefore, the proposed system can be used to classify reliably and precisely several cell types with different intrinsic physical properties for personalized cancer medicine development.

## Introduction

Cell separation from a heterogeneous mixture of cells is critical for cancer research and new personalized drug development^1–7^. The precise isolation of distinct cell types provides a better understanding of cellular functions and roles in biological systems, and enables the identification of specific cell populations involved in disease progression and treatment response^1–12^. Cell separation techniques have been developed based on cell-surface markers, such as fluorescent dyes^13–15^ and specific antibodies^16,17^ or intrinsic physical cell properties, including size, density, and compressibility^18–21^. Among these techniques, label-free cell sorting methods based on intrinsic physical biomarkers have been widely used because they do not require intensive tasks or specific cell-surface labels to identify cells of interest. Thus, unwanted side effects on normal cell physiology and activity can be minimized compared with those of conventional label-aided cell sorting methods, such as fluorescent-activated cell sorting and magnetic-activated cell sorting^18,19^. Approaches such as optical tweezers and microfluidic platforms can effectively and reliably separate cells. However, these methods suffer from critical limitations such as photothermal effect along with the use of a strong light intensity, difficult techniques, and undesirable effects of shear stress, stiction, and blockage on cellular functions and responses owing to structural irregularities within microstructures^20–23^.

Ultrasound-based acoustic tweezers have recently been demonstrated to be capable of capturing single cells or measuring physical cell properties as a backscattering coefficient with a relatively simple and cost-effective experimental setup^24–27^. Longer ultrasound pulses and subsequent short pulses are required to securely manipulate and acquire backscattered signals from the trapped single cell, respectively, using either the same transducer or different transducers for each procedure. However, precise measurement in a trapped single cell is challenging owing to the inevitable use of two different pulse sequences along with the experimental setups, which may result in misleading information. To address this critical limitation, acoustic tweezers with high-frequency ultrasound at a high pulse repetition frequency (PRF) were developed to simultaneously trap a targeted single cell and measure its backscattered signals^28^. Monocycle ultrasound pulses at a high PRF are capable of trapping a targeted single cell, with a lower level of acoustic trapping force compared with that of conventional acoustic tweezers with excessive acoustic energy with longer pulses. Moreover, they can simultaneously measure the backscattered signals from the trapped single micron-sized objects, to identify two different microbead diameters, such as 5 and 10 $\upmu$m, and two different cell diameters, including red blood cells (RBCs) with diameters between 6 and 8 $\upmu$m and normal SV40 immortalized epithelial prostate (PNT1A) cells with diameters between 9 and 11 $\upmu$m, without compromising cell viability. However, postprocessing of the integrated backscattering (IB) coefficients based on measured backscattered signals is typically a time-consuming process and causes possible errors because of the manual setting of reflected signal time between the first and tiny reflected ultrasound signal produced by the trapped single object. Moreover, the huge reflected ultrasound signal comes from the thin Mylar film, as the IB coefficient is defined as the ratio of the backscattered energy from a scatterer volume to that from a flat quartz target. To overcome the limitations caused by manual analysis, deep learning-empowered artificial intelligence models are employed in this study to minimize the postprocessing.

Several approaches can be used to analyze the characteristics of live cells, including heuristic-based manual analysis, conventional machine learning (ML) methods, and deep learning models. First, heuristic approaches are simple and intuitive, but have inherent limitations. Further more, conventional ML algorithms do not have sufficient capabilities to express correlations of cell characteristics with observed data. Finally, state-of-the-art (SOTA) deep learning models have high expressive capabilities but require a large amount of training data that is difficult to collect from live cells. In our previous studies, we demonstrated that relatively shallow convolutional neural network (CNN) models can be an effective and efficient solution^29,30^ to investigate the physical properties of cells, such as cell stiffness and structure of the cell membrane by comparing their microscopic images before and after inflicting the high-frequency ultrasound beam. Using VGG (Visual Geometry Group)-like^31^ CNN models as backbones, breast cancer cells were successfully classified based on invasiveness and approximated Young’s moduli of cells with high accuracy. Inspired by our previous studies, we focused on determining whether neural network models can identify and separate micron-sized single objects based on patterns of backscattered signals from targeted objects.

In this study, we propose an approach for automated cell-type classification by improving some aspects of the current postprocessing pipeline, along with label-free acoustic sensing of a trapped single object. Our CNN models can discover cell physical properties by analyzing backscattered signals. Additionally, we expect the CNN models to be robust to noise on raw signals, such as signals reflected from surrounding objects. For the experiments, we collected backscattered signals using a label-free single-cell analysis system^28^, denoised the raw backscattered signals using CNN autoencoders, and classified cells into their cell types by analyzing the denoised signals with CNN backbones and fully connected neural networks.

Furthermore, although a previous study^28^ showed that cell diameters affect their backscattered signals, further investigation is required to explore how other cell aspects may lead to differences in backscattered signals. Thus, we attempted to reveal other cell characteristics that influence backscattered signals and whether neural network models can capture these characteristics. The denoised signals were transformed into waveform signals, Fourier spectra, and spectrograms. Subsequently, we used the proposed CNN backbones to extract features in the time, frequency, and time-frequency domains from the transformed signals. Because each backbone focuses on the respective features of backscattered signals, we can assume the type of cell properties that causes differences in signal characteristics by examining the performance of the backbones in classifying cells and polystyrene microspheres, according to their types and physical properties. Therefore, we evaluated the proposed system based on the following research questions:

- RQ 1. Cells have distinctive patterns of backscattered signals according to their type;
- RQ 2. Frequency spectrum analysis is useful to discover characteristics of backscattered signals;
- RQ 3. Temporal changes in the frequency spectra are significant for analyzing the patterns of backscattered signals.

RQ 1 has been validated by the performance of the proposed neural network models for automated cell-type classification (Table 1). We conducted more detailed experiments to examine the cell characteristics that affect the backscattered signals as follows: distinguishing (1) cell types with different diameters (Table 1), (2) polystyrene microspheres with different diameters (Table 2), and (3) micron-sized objects with different physical properties and similar diameters (Table 3). We have verified RQ 2 by comparing the performance of the time domain analysis (i.e., applying one-dimensional (1D) CNN to raw signals) with that of the frequency domain analysis (Tables 1, 2, 3). Moreover, we compared the spectrogram with frequency spectrum analyses to validate RQ 3 (Tables 1, 2, 3 and Fig. 5). Additionally, we demonstrated that noise significantly hinders the performance of the proposed models and should be addressed using ablation tests (Table 4).

**Table 1:** Performance of the proposed model in waveform, frequency spectrum, and spectrogram. A, $F_1$, P, and R indicate accuracy, $F_1$ score, precision, and recall, respectively. Avg. and S.D. refer to the mean and standard deviation of three folds, respectively.

PNT1A/RBC	Waveform				Frequency Spectrum				Spectrogram			
	A	$F_1$	P	R	A	$F_1$	P	R	A	$F_1$	P	R
1-Fold	0.97	0.98	1.00	0.96	0.96	0.97	0.97	0.97	0.98	0.99	1.00	0.97
2-Fold	0.97	0.98	0.97	1.00	1.00	1.00	1.00	1.00	1.00	1.00	1.00	1.00
3-Fold	0.97	0.64	0.63	0.67	1.00	1.00	1.00	1.00	1.00	1.00	1.00	1.00
**Avg.**	0.98	0.88	0.88	0.88	0.99	0.99	0.99	0.99	**0.99**	**1.00**	**1.00**	**0.99**
**S.D.**	0.02	0.20	0.22	0.18	0.02	0.02	0.02	0.02	**0.01**	**0.01**	**0.00**	**0.02**
SVM	0.76	0.76	0.76	0.76	0.86	0.86	0.86	0.86	0.96	0.96	0.96	0.96
Logit	0.74	0.74	0.74	0.75	0.78	0.78	0.79	0.79	0.96	0.96	0.96	0.96
MLP	0.78	0.78	0.78	0.78	0.92	0.92	0.92	0.92	0.51	0.51	0.51	0.51

Models with the highest evaluation metrics are highlighted in [bold].

**Table 2:** Performance of the proposed model in waveform, frequency spectrum, and spectrogram to classify polystyrene microspheres (i.e., cell-mimicking beads) according to their sizes.

5 $\upmu$m/10 $\upmu$m	Waveform				Frequency Spectrum				Spectrogram			
	A	$F_1$	P	R	A	$F_1$	P	R	A	$F_1$	P	R
1-Fold	1.00	1.00	1.00	1.00	1.00	1.00	1.00	1.00	1.00	1.00	1.00	1.00
2-Fold	1.00	1.00	1.00	1.00	1.00	1.00	1.00	1.00	1.00	0.95	0.95	0.95
3-Fold	1.00	1.00	1.00	1.00	1.00	1.00	1.00	1.00	0.99	0.99	0.99	1.00
Avg.	**1.00**	**1.00**	**1.00**	**1.00**	**1.00**	**1.00**	**1.00**	**1.00**	1.00	0.98	0.98	0.98
S.D.	**0.00**	**0.00**	**0.00**	**0.00**	**0.00**	**0.00**	**0.00**	**0.00**	0.00	0.03	0.02	0.03

Significant values are in [bold].

**Table 3:** Performance of the proposed models for distinguishing cells from similar-sized polystyrene microspheres (i.e., cell-mimicking beads). The upper and lower parts of the table present results between PNT1A ($10.10 \pm 0.88\;\upmu$m) and 10 $\upmu$m microspheres ($9.97 \pm 0.07\;\upmu$m) and results between RBC ($6.57 \pm 0.66\;\upmu$m) and 5 $\upmu$m microspheres ($4.98 \pm 0.06\;\upmu$m), respectively.

	Waveform				Frequency Spectrum				Spectrogram			
	A	$F_1$	P	R	A	$F_1$	P	R	A	$F_1$	P	R
**PNT1A/10 **$\upmu$**m**												
1-Fold	0.97	0.64	0.67	0.61	0.97	0.64	0.67	0.61	1.00	1.00	1.00	1.00
2-Fold	1.00	1.00	1.00	1.00	1.00	1.00	1.00	1.00	0.98	0.73	0.71	0.75
3-Fold	1.00	1.00	1.00	1.00	1.00	1.00	1.00	1.00	1.00	1.00	1.00	1.00
**Avg.**	0.99	0.88	0.89	0.87	0.99	0.88	0.89	0.87	**0.99**	**0.91**	**0.90**	**0.92**
**S.D.**	0.02	0.21	0.19	0.22	0.02	0.21	0.19	0.22	**0.01**	**0.16**	**0.16**	**0.14**
**RBC/5** $\upmu$**m**												
1-Fold	1.00	1.00	1.00	1.00	1.00	1.00	1.00	1.00	1.00	1.00	1.00	1.00
2-Fold	1.00	1.00	1.00	1.00	1.00	1.00	1.00	1.00	1.00	1.00	1.00	1.00
3-Fold	1.00	1.00	1.00	1.00	1.00	1.00	1.00	1.00	1.00	1.00	1.00	1.00
**Avg.**	**1.00**	**1.00**	**1.00**	**1.00**	**1.00**	**1.00**	**1.00**	**1.00**	**1.00**	**1.00**	**1.00**	**1.00**
**S.D.**	**0.00**	**0.00**	**0.00**	**0.00**	**0.00**	**0.00**	**0.00**	**0.00**	**0.00**	**0.00**	**0.00**	**0.00**

Significant values are in [bold].

**Table 4:** Performance of the proposed denoising autoencoder model in distinguishing RBC and PNT1A cells. Raw, DN, and DN+ indicate the case without using any denoising method, the case only using denoising autoencoder, and the case using denoising autoencoder and injecting the Gaussian noise, respectively.

	Waveform				Frequency scpectrum				Spectrogram			
	A	$F_1$	P	R	A	$F_1$	P	R	A	$F_1$	P	R
Raw	0.99	0.88	0.89	0.88	0.98	0.88	0.88	0.88	0.99	0.99	1.00	0.99
DN	0.98	0.88	0.88	0.88	0.98	0.88	0.86	0.89	0.99	1.00	0.99	**1.00**
DN+	0.98	0.88	0.88	0.88	0.99	0.99	0.99	0.99	**0.99**	**1.00**	**1.00**	0.99

Significant values are in [bold].

The remainder of this paper is organized as follows. The “Materials and methods” section describes the label-free single-cell analysis system used for collecting backscattered signals from live cells, the proposed autoencoder model for denoising raw backscattered signals, and the proposed CNN backbones for discovering cell properties from backscattered signals. In the “Results” section, we present experimental procedures and results to evaluate the proposed classification system and validate our research questions. The discussion, concluding remarks, and future research directions are presented in the “Discussion and concluding remarks” section.

## Materials and methods

Backscattered signals of two types of cells (PNT1A and RBC) and polystyrene microspheres (5 $\upmu$m and 10 $\upmu$m) were collected using a label-free single-cell analysis system^28^. We applied the denoising autoencoder and classifiers based on artificial neural networks to validate our assumption, that is, the backscattered signal is significantly distinctive in distinguishing particular types of cells from the others. The backscattered signals were denoised by the proposed autoencoder model, and our CNN classifiers were trained to discover cell properties from the time domain or frequency domain analysis.

### Experimental setup for label-free single-cell isolation and analysis

**Figure 1:**
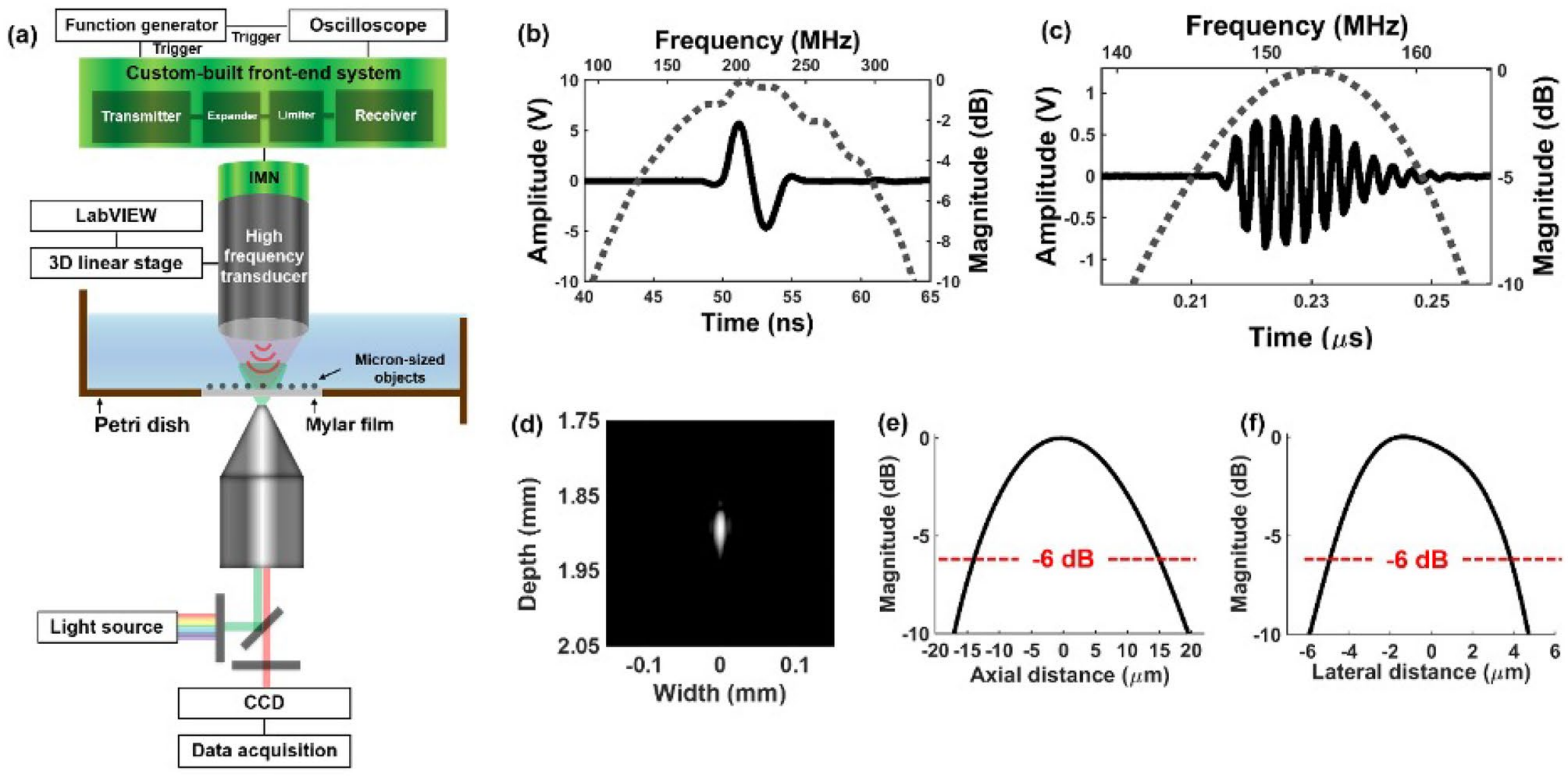
Label-free single-cell separation and analysis system. (**a**) Experimental setup consisting of a high-frequency ultrasound transducer with impedance matching network, custom-built front-end system, a three-dimensional linear stage, and oscilloscope, and an inverted fluorescence microscope with an image acquisition and analysis tool. (**b**) Measured pulse waveform and its spectrum of the custom-built front-end system. (**c**) Measured pulse-echo waveform and its spectrum of high-frequency ultrasound transducer with impedance matching network. (**d**–**f**) Measured spatial ultrasound pressures of the high-frequency ultrasound transducer with impedance matching network.

Figure 1a demonstrates experimental setup for label-free single-cell isolation and analysis composed of a custom-built tightly focused high-frequency ultrasound transducer operated with its impedance matching network along with custom-built front-end system, a three-dimensional (3D) linear stage, an oscilloscope, and an inverted fluorescence microscope with an image acquisition and analysis tool^28^. The lithium niobate ($LiNbO_3$)-based highly focused high-frequency transducer with an aperture size of 2.6 mm and focus number of 0.75 was designed and fabricated according to the transducer fabrication process^32^. The attachable impedance matching network was developed to maximize energy transfer efficiency between the transducer and the custom-built front-end system^33^. The location of the custom-built high-frequency transducer with the impedance matching network was precisely adjusted by 3D linear stage (SGSP 20, Sigma KOKI Co., Japan) controlled by a customized LabVIEW (National Instruments, Austin, TX, USA) program. A custom-built front-end system developed in compact and cost-effective printed circuit board (PCB) board was comprised of a transmitter for generating high-frequency ($\ge 100$ MHz) and high-PRF ($\le 1$ MHz) monocycle bipolar pulses, a receiver with an enhanced signal-to-noise ratio for amplifying considerably weak backscattering signals, and diode-based expander and limiter for protecting the transmitter and receiver, respectively^28,34–36^. The backscattered signals from the trapped single object on the acoustically transparent Mylar film were recorded with sampling rate of 10 GHz using the oscilloscope (104MXi, LeCroy, Santa Clara, CA, USA). The inverted fluorescence microscope (IX71, Olympus, Center Valley, PA, USA) with the image acquisition and analysis tool (Metamorph, Molecular Devices, Sunnyvale, CA, USA) were used to acquire time-resolved bright-field images for demonstrating high-frequency ultrasound pulse-induced trapping and moving single object such as particle or cell.

### Performance of front-end system, high-frequency ultrasound transducer with impedance matching network, and acoustic trapping

Figure 1b represents measured performance of the front-end system capable of generating monocycle bipolar pulses with a center frequency of 200 MHz and a $-6$ dB bandwidth of 110–290 MHz. The performance of the developed high-frequency ultrasound transducer with its impedance matching network was measured by a pulse-echo response with a flat quartz and wire-phantom imaging with a 2.5 $\upmu$m diameter tungsten wire in degassed and deionized water. From the pulse–echo response in Fig. 1c, the measured center frequency and $-6$ dB bandwidth of the high-frequency ultrasound transducer with the impedance matching network were 153 MHz and 144–162 MHz, respectively. The measured axial and lateral dimensions of the transducer focus were 28.5 and 8.6 $\upmu$m, respectively, defined by the full width at half maximum (FWHM, $-6$ dB pressure) of the pressure field based on the wire-target image as presented in Fig. 1d–f.

**Figure 2:**
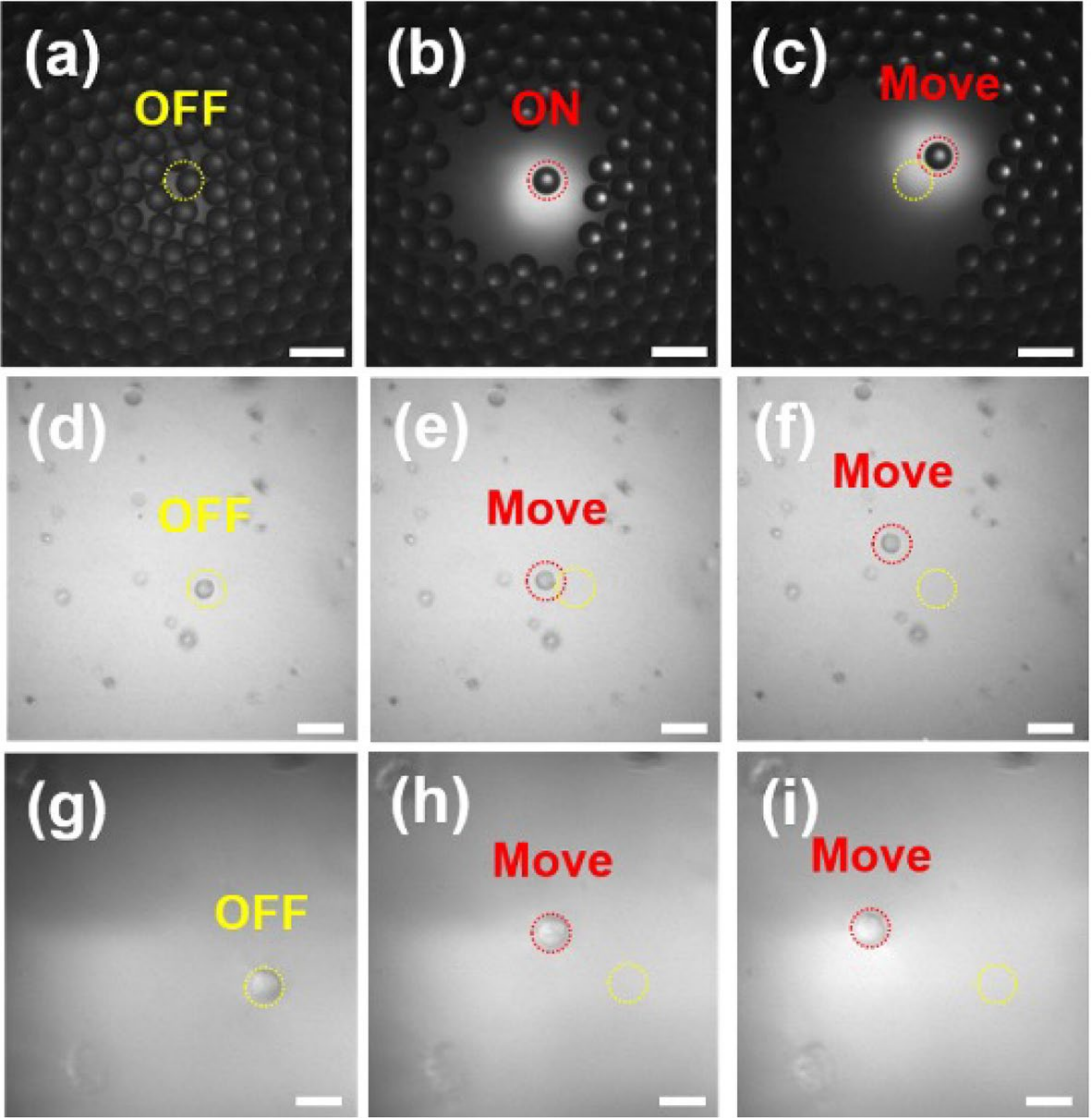
Acoustic trapping of (**a**–**c**) the targeted single polystyrene microsphere, (**d**–**f**) RBC, and (**g**–**i**) PNT1A cell with movement of the high-frequency ultrasound transducer. Yellow and red dashed circles indicate the initial and moved locations of the transducer, respectively. Scale bars in the images indicate (**a**–**c**) 100 $\upmu$m and (**d**–**i**) 20 $\upmu$m.

In addition, the capability of monocycle ultrasound pulses at high PRF for trapping targeted single object was demonstrated in Fig. 2. Acoustic trapping force calibration system was developed with a pressure controller (ez-gSEAL 100B, Neobiosystem, CA, USA), a glass capillary with filament (GD-1, Narishige, NY, USA), and a vertical micropipette puller (PC-10, Narishige, NY)^37–39^. Measured acoustic trapping force of $122.4 \pm 13.4$ nN ($n = 3$) generated by monocycle electrical pulses with pulse length of 6.7 ns and applied input voltage of 50 V at a PRF of 167 kHz enabled to simultaneously capture and move microsphere, RBC, and PNT1A cell along with the direction of the transducer, and acquire backscattered signals from the trapped single object.

### Cell preparation

Fresh human blood samples were obtained from healthy volunteers providing the informed consent form. All experiments with the obtained blood were conducted in accordance with the guidelines and regulations, which was approved by the institutional review board (IRB) of the University of Southern California (UP-16-00713). The collected whole blood was centrifuged with phosphate-buffered saline (PBS) (Thermo Fisher Scientific, Waltham, MA, USA) at 500*g* for 10 min to separate RBCs. After gently eliminating the supernatant, the RBCs in PBS were centrifuged again and resuspended in a mixed solution of PBS and Alsever’s solution. PNT1A cells (Sigma-Aldrich, St. Louis, MO, USA) cultured in RPMI 1640 supplemented with 10$\%$ fetal bovine serum were grown as a monolayer in a 37 $\circ$C incubator with a humidified atmosphere of 5$\%$ CO$_2$. The PNT1A cells were gently washed twice with PBS, dispensed with TrypLE solution in an incubator for 5 min, and centrifuged at 150$\times$*g* for 5 min to separate them. After gently eliminating the supernatant, PNT1A cells in PBS were centrifuged again and resuspended in Dulbecco’s PBS with Ca$^{2+}$ (Thermo Fisher Scientific, Waltham, MA, USA).

### Denoising backscattered signals and data augmentation

As it was difficult to precisely capture the backscattered signals from cells within signals from surrounding objects such as mylar film, we initially conducted the process of recovering backscattered signals from cells buried under reflections from other surrounding objects. Recently, denoising autoencoder models have been widely employed to solve this problem^40,41^. An autoencoder, which is an artificial neural network architecture, compresses features extracted from input data into low-dimensional feature vectors (or matrices/tensors) and recovers the original input from the feature vectors. Owing to the dimensionality of the feature vectors, the autoencoder cannot recover all details of the input, and only distinctive characteristics remain in its output.

Recurrent neural network (RNN) models, which consider the $n+1$-th input and *n*-th output to generate the $n+1$-th output, have been widely used for analyzing sequential data. Although the RNN mechanism is effective in learning temporal correlations between input samples, it also causes an inherent limitation called the long-term dependency problem^42^. Owing to this problem, earlier inputs are given less preference than later ones and are finally forgotten. Because cell characteristics are not reflected by the last few samples of backscattered signals, models that can analyze all samples together are required. Therefore, we used a 1D convolutional autoencoder for denoising. For every sample in the signals, 1D convolution operations accumulate information on the temporally adjacent samples. Although each convolution filter focuses on the local context, we can extract the global features of signals by stacking the convolution layers. Thus, the 1D convolutional autoencoder can overcome limitations of the recurrent autoencoder, and it is more suitable for sequential data than 2D convolutional or fully connected autoencoder models. Moreover, we employed dilated convolution to efficiently discover global features from the signals.

Dilated convolution layers extend the receptive fields of conventional operations that observe only adjacent pixels or samples. Thus, when two samples that are temporally distant have correlations, we require numerous layers to propagate information from one sample to the other. Dilated convolution can solve this problem by extending the receptive fields of convolution operations. Observing wider ranges at a time enabled us to analyze the correlations between distant samples while reducing the number of layers. Samal et al.^43^ proposed a 1D convolutional autoencoder model that replaces down/up sampling layers with convolutional layers. This approach makes receptive fields wider but computationally expensive. Our experimental results in Table 4 show that the current number of parameters is sufficient for learning the characteristics of backscattered signals of cells.

**Figure 3:**
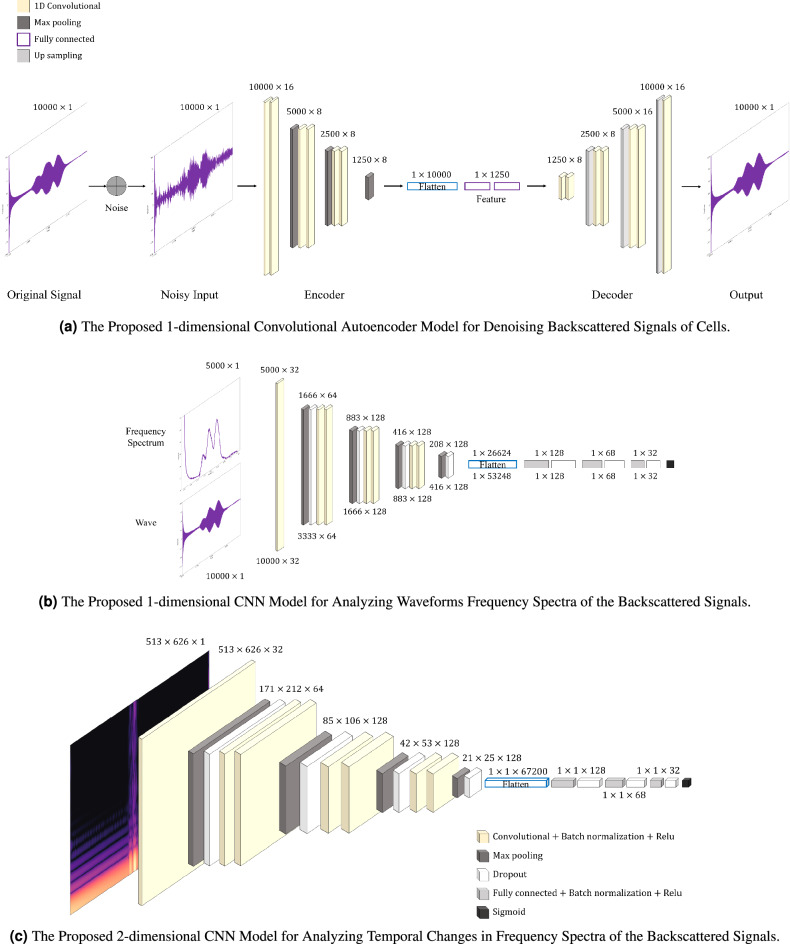
Structures of the proposed CNN models. The proposed system consists of three main components: the high-frequency ultrasound transducer in Fig. 1, denoising autoencoder in (**a**), and CNN classifiers in (**b**) and (**c**). The denoising autoencoder reduces noise and emphasizes latent features in backscattered signals collected by the high-frequency ultrasound transducers. The denoised signals were processed by FFT or STFT. In the waveform analysis, we did not conduct additional processing of the denoised signals. Then, the signals (or spectra/spectrograms) become inputs to the CNN models to classify cells according to cell type.

**Figure 4:**
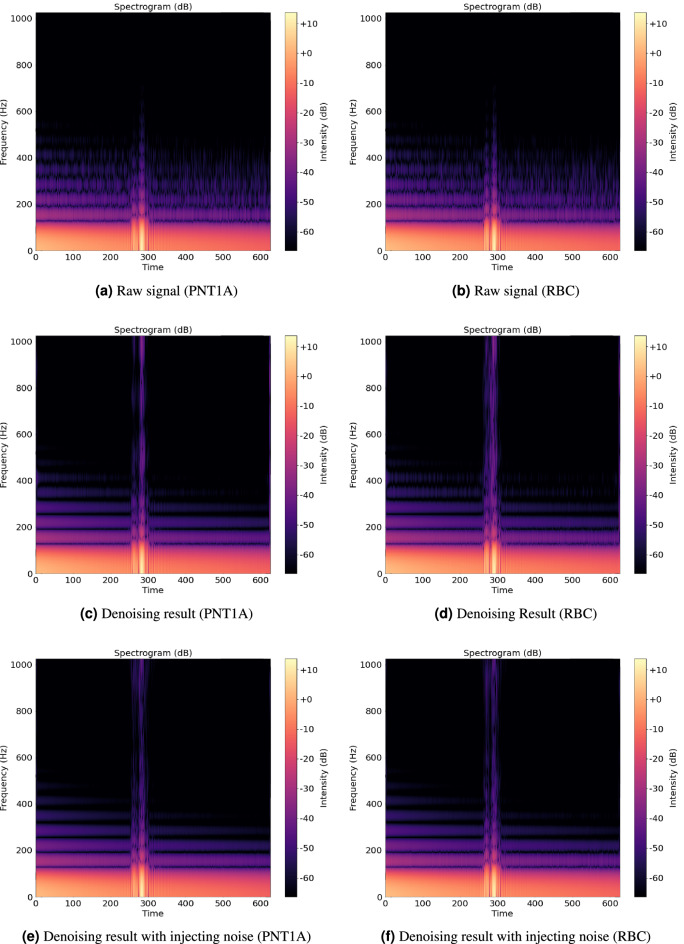
Results of the proposed denoising autoencoder models. Figures on the left side are spectrograms of a PNT1A cell, and spectrograms of an RBC are on the right side. The X and Y-axes indicate time and frequency, respectively. Furthemore, the brightness of colors refers to the intensity of the frequency components. The time unit corresponds to 16 samples in the signal, and the units of frequency and intensity are *Hz* and *dB*, respectively. (**a**) and (**b**) are the spectrograms extracted from the original signal, (**c**) and (**d**) are based on our denoising autoencoder using 1D CNN, and (**e**) and (**f**) are cases in which the denoising autoencoder and Gaussian noise injection were used together.

As shown in Fig. 3a, the encoder part of the proposed autoencoder model consists of six dilated-1D convolution layers, three max pooling layers, and two fully connected layers. From the compressive feature vectors *Z* generated by the encoder, the decoder restores the original signals using seven dilated-1D convolution layers and three upsampling layers. This can be formulated as follows:

1 $$\begin{aligned}&{Z} =f(X;W_e),~~ \hat{X}=g(Z;W_d),\\&W_e^*,W_d^* = \mathop {\hbox {argmin}}\limits _{W_e,W_d} \Vert X - \hat{X} \Vert _2^2. \nonumber \end{aligned}$$

where *X* is the raw signal acquired from the cells, $\hat{X}$ is the denoised signal restored from the feature vectors, and $W_e$ and $W_d$ denote the parameter sets of the encoder and decoder, respectively. $f(\cdot ;\cdot )$ and $g(\cdot ;\cdot )$ represent the encoder and decoder, respectively. The activation function of the layers in the proposed autoencoder was rectified linear unit (ReLU), and the mean absolute error (MAE) loss and Adam optimizer^44^ were employed to train the autoencoder. Figure 4a–d show that the proposed autoencoder recovers high-frequency features in the raw signals. However, employing only a denoising autoencoder does not significantly contribute to the accuracy of cell classification, as shown in Table 4.

Gaussian noise injection can enhance the robustness of the proposed system to noise and surroundings at the stage of noise reduction, and classification^45^. As shown in Fig. 3a, the original signal *X* is corrupted to a noisy signal $\tilde{X}$ by a stochastic mapping $\tilde{X} \sim q(\tilde{X} \mid X)$ with Gaussian noise $n \sim N(0, \sigma _n^2)$. The corrupted signal was used as the input data to the encoder for extracting the feature *Z*, and to obtain the reconstructed denoising result $\hat{X}$, as follows:

2 $$\begin{aligned} {Z} =f(\tilde{X};W_e) =f(X+n;W_e),~~ \hat{X}=g(Z;W_d). \end{aligned}$$

Furthermore, because noise is randomly generated, we can extend the number of input signals. Through this data augmentation process, the autoencoder can be trained to distinguish the distinctive patterns of backscattered signals from redundant noise. We applied this augmentation method to denoising and classifiers. Figure 4 shows examples of raw signals collected from RBC and PNT1A cells, the results of the proposed autoencoder, and cases in which Gaussian noise is injected into the inputs of the autoencoder.

### Automated cell classification based on convolutional neural networks

This study did not employ sophisticated neural network architectures to classify the backscattered signals of cells into cell types. Despite the high performance of SOTA deep learning models, it is challenging to collect large-scale data, which are required to train these models from live cells. As demonstrated in our previous studies^29,30^, relatively shallow neural network models can be an efficient and effective solution for analyzing live cells with a limited amount of data. The structures of our CNN models are similar to those of VGG16^31^ but employ techniques for extending receptive fields and preventing overfitting. Patterns of backscattered signals of cells were not as distinctly visible to the naked eye in both the time and frequency domain signals (Fig. 4). However, the proposed CNN models effectively recovered patterns that were buried under noise.

In VGG16^31^, fixing the size of the convolutional kernel to $3\times 3$ and increasing the depth of the network improves the classification accuracy. Performing multiple convolutions with a small filter reduces the number of parameters compared with employing a few convolution layers with large filters. This approach enabled us to increase the training efficiency and reduce the risk of overfitting. Simultaneously, as the number of layers increased, the CNN model could extract nonlinear features from broader areas of the input data.

However, extracting features from adjacent samples in the backscattered signals causes difficulties in capturing the global characteristics of the signals. To address this problem, Lu et al.^46^ improved Gliomas classification performance by using ResNet^47^ with dilated convolution^48^, which can obtain a larger receptive field without increasing the size of the convolution kernels. Chen et al.^49^ proposed a lung segmentation method that combines U-Net^50^ and dilated convolution in computed tomography (CT) images. We applied VGG-like models fused with dilated convolution to the backscattered signals of the cells to extend the receptive fields of the convolution filters. Thus, we obtained signal features that considered both the local and global perspectives with only a small number of convolution layers and parameters.

Furthermore, the input distribution of each layer changed because the parameters of the former layers were updated based on epochs. Therefore, as neural network models deepened, the input distribution fluctuated significantly, making parameter learning unstable. Therefore, we applied batch normalization^51^ to each layer, which normalizes the output of the layers to improve learning speed and efficiency. Furthermore, we employed regularization and initialization of weights, biases, and dropout layers to increase the training efficiency and prevent overfitting. The detailed architecture of the proposed neural network models is presented in the remainder of this section.

#### Waveform and frequency spectrum analysis

We applied the same 1D CNN classifier to analyze the waveforms and frequency spectra of the denoised backscattered signals of the cells. The 1D CNN classifier consisted of seven dilated 1D convolution layers, three fully connected layers, seven dropout layers, and four max pooling layers, as shown in Fig. 3b. Additionally, we conducted batch normalization for each convolution layer, and L2 regularization and Xavier normal initialization^52^ were applied to both the weights and biases of the convolution and fully connected layers.

Existing studies^28^ on methods for collecting backscattered signals from cells surmised that the sizes of cells/particles affect the frequency spectra of the backscattered signals. Furthermore, as described in the previous section, a label-free single-cell analysis system inflicts high-frequency ultrasound microbeams on cells over a significantly short time. Thus, the signal backscattered from the cells is also significantly short compared with the entire signal, as shown in Fig. 4. If we analyze the waveform directly, searching for a short period, including substantive signals from cells, can be an additional burden. However, in the frequency spectrum analysis, we did consider this point. Therefore, we expected to perform the frequency spectrum analysis with higher and more stable accuracy than the waveform analysis.

Using fast Fourier transform (FFT)^53^, we extracted the frequency spectra of the backscattered signals, which were denoised by the proposed autoencoder model. For a discrete signal $\hat{X}(n)$ where $n \in [0, N-1]$, and *N* is the number of samples, its frequency spectrum $\hat{X}(f)$ and FFT can be formulated as:

3 $$\begin{aligned} \hat{X}(f) = \sum _{n=0}^{N-1} \hat{X}(n) \cdot e^{-i\frac{2\pi f}{N}n}, \end{aligned}$$

where $i=\sqrt{-1}$ is an imaginary unit, and $f \in [0, N-1]$corresponds to the frequency components. Thus, $\hat{X}(f)$ indicates the amplitude of frequency *f* in $\hat{X}(n)$. Because $\hat{X}(f)$ is a symmetric function, we only use its right half.

Both the frequency and waveform signals were sequential data. However, we could control the full length of the signals, which is much longer than the actual backscattered signals from the cells, and later samples or higher-frequency components were not more significant than earlier samples or lower-frequency components. Therefore, we extracted local features of signals in the time and frequency domains using a 1D CNN rather than an RNN, which can cause the volatilization of earlier inputs. The fully connected layers were then trained to discover differences in the local features based on the cell types. Figure 3b shows the detailed structure of the proposed 1D CNN classifier. In this study, we evaluated the proposed system by distinguishing cancer cells in the blood (PNT1A) from RBC. Thus, we used sigmoid activation on the output layer and binary cross-entropy loss. To further increase the robustness of the system to noise and prevent overfitting, we conducted data augmentation using Gaussian noises, as with the proposed autoencoder. For the frequency spectrum analysis, we first injected Gaussian noise and then conducted an FFT. For both the frequency spectra and waveform signals, the 1D CNN classifier can be formulated as:

4 $$\begin{aligned}&\hat{Y_i} =h_{1D}(\hat{X_i} + n;W_c),\\&W_c^* = \mathop {\hbox {argmin}}\limits _{W_c} - \sum _{\forall \hat{X_i}} \left( Y_i \log {\hat{Y_i}} + \left( 1-Y_i \right) \log \left( 1- \hat{Y_i} \right) \right) , \nonumber \end{aligned}$$

where $Y_i$ and $\hat{Y_i}$ are the actual and predicted cell types, respectively, that correspond to the *i*-th input signal $X_i$, $h_{1D}(\cdot ;\cdot )$ represents the proposed 1D CNN model, and $W_c$ denotes a parameter set of the model.

#### Spectrogram analysis

The amplitudes of the frequency components and temporal changes in the amplitudes were revealed in the frequency spectra and waveform signals, respectively. Time-frequency domain transformation can be employed to analyze the features of the frequency and amplitude simultaneously. We expect that this integration, called a spectrogram, can describe various characteristics of cells better than 1D representations. The spectrogram represents the intensity of the signals in the time-frequency plane (2D space), as shown in Fig. 4. For the transformation, we employed a short-term Fourier transform (STFT)^54^ that consecutively conducts FFT for part of a signal using a fixed-length sliding window. When the window size was l and the step size was d, we first conducted FFT for a signal $\langle \hat{X}(0), \ldots , \hat{X}(l-1) \rangle$, and on the next iteration the window moved to $\langle \hat{X}(d), \ldots , \hat{X}(d+l-1) \rangle$ (in this study, $l=32$ and $d=16$). Consequently , we obtained the frequency spectra for each time window. This can be formulated as follows:

5 $$\begin{aligned} \hat{X}(f,m) = \sum _{n=-\infty }^{\infty } \hat{X}(n) \cdot w(f - n) \cdot e^{-i\frac{2\pi m}{N}n}, \end{aligned}$$

where *w*(*n*) is the window function, and *m* is the discrete frequency variable. The raw signal was divided into nine segments with $50\%$ overlap; each segment was windowed with a Hamming window, and the sample rate was 1,000,000 Hz. The spectrogram is the squared magnitude of STFT.

Zhu et al.^55^ applied a 1D CNN to the frequency domain analysis of spectrograms and long short-term memory (LSTM) to analyze temporal changes in the frequency spectra. This convolution RNN approach has advantages in representing the temporal characteristics of data in an explicit manner. However, the recurrent layers cause long-term dependency problems. Thus, in this study, we applied dilated 2D convolution to the spectrograms to capture the characteristic local structure in which the cell type is distinguished from the entire signal.

Because we analyzed the same problem of classifying cell types in the time-frequency domain, a 2D CNN model was constructed by extending the number of dimensions in the structure of the 1D CNN model presented in the previous section. The main difference between the 2D CNN classifier and the 1D CNN is the number of parameters resulting from the increased number of dimensions, while their structures are almost identical.

Figure 3c shows the architecture of the proposed 2D CNN classifier in detail. For the binary classification of cell types, the activation function on the output layer and the loss function are the sigmoid activation and binary cross-entropy loss, respectively. Before applying the SFTF to raw signals, we conducted data augmentation using Gaussian noises to address the robustness of the classifier to noise and overfitting, as with the 1D CNN classifier. Therefore, the 2D CNN classifier for the spectrogram of signals can be formulated in a manner similar to Eq. (4).

## Results

**Figure 5:**
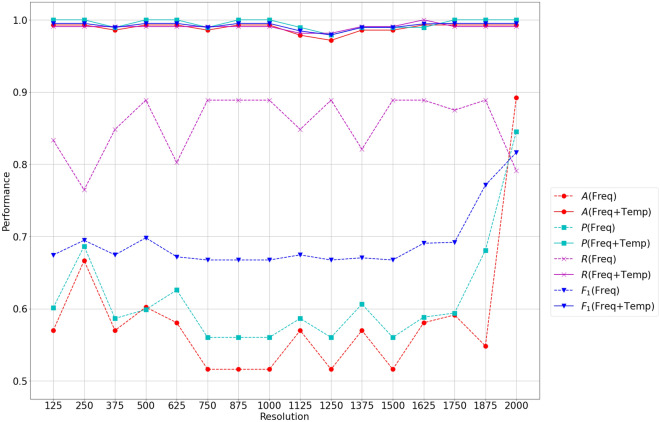
Performance evaluation of the proposed model in terms of accuracy (A), precision (P), recall (R), and $F_1$ measure ($F_1$) as a function of frequency resolution. Solid and dotted lines indicate the results of training the signal in the frequency spectrum (Freq) and spectrogram (Freq+Temp), respectively.

We validated our research questions by applying the proposed system to classify live cells (RBC and PNT1A). As a combination of the existing ultrasound equipment (i.e., label-free single-cell analysis system) and conventional CNN models, the significance of the proposed system comes from automating cell-type classification by analyzing the backscattered signals of cells. Therefore, we first validated the distinctness of the backscattered signals (RQ 1) by examining whether the proposed system is capable of distinguishing live cells from other cells or particles (Tables 1, 2, 3). In the signal analysis, we presented three approaches (waveform, frequency spectrum, and spectrogram analysis) based on the preprocessing of the signals. These approaches have advantages and disadvantages in terms of the computational complexity of preprocessing and model training. Thus, we attempted to verify whether additional preprocessing contributed to the performance of the entire system (RQ 2 and RQ 3). This verification also revealed the significance of the frequency and time domain features of the signals backscattered from the cells (Fig. 5). Additionally, as shown in Fig. 4, the collected signals were noisy, and our period of interest was a significantly short pulse. Therefore, we evaluated the robustness of the proposed system to noise and the contribution of the proposed autoencoder to system performance by conducting ablation tests for denoising techniques (Table 4).

### Experimental settings

This section describes the experimental setup, including the datasets, evaluation metrics, hyperparameter settings, and baseline methods. We collected 77 signals for each cell type (12 RBC and 12 PNT1A cells) and 146 signals for polystyrene microspheres (72 signals obtained from 17 microspheres of 5 $\upmu$m and 74 signals measured from 14 microspheres of 10 $\upmu$m). The mean and standard deviation of the size of RBC and PNT1A cells were $6.57 \pm 0.66\;\upmu$m and $10.10 \pm 0.88\;\upmu$m, respectively, and those of 5 $\upmu$m and 10 $\upmu$m microspheres were $4.98 \pm 0.06\;\upmu$m and $9.97 \pm 0.07\;\upmu$m, respectively. The backscattered signals of single object such as microsphere or cell were measured in the acoustically trapped state by ultrasound. Because they were simultaneously trapped and measured, the signal could be obtained from only targeted object rather than information from the surrounding objects, which could be the main advantage of this technology. However, these complex experimental procedures also cause difficulties in obtaining large data sets. A small data set is an intrinsic limitation of the domain of single-cell analysis using acoustic tweezers^56–62^, which may lead to overfitting issues and data reliability of experimental results. To address possible issues, we initially conducted 3-fold cross-validation to examine whether the proposed model could deal with the diversity of live cells. In each experiment, we divided our dataset into three equal parts, while preserving the distributions of labels (e.g., types of cells and microspheres). To ensure that every backscattered signal was used as a testing sample at least once, each validation case employed two of the three parts of the dataset as training data and the remaining one part as testing data. Subsequently, we augmented the dataset by injecting Gaussian random noise. We generated ten noisy signals for each original backscattered signal in the training data.

We used four evaluation metrics: accuracy (A), precision (P), recall (R), and $F_1$ measure ($F_1$). When *T* indicates a set of automatically detected PNT1A cells and $T^*$ refers to a set of actual PNT1A cells, the accuracy can be formulated as:

6 $$\begin{aligned} A = \frac{|T^* \cap T |+ |(T^* \cup T)^c |}{|U |}, P = \frac{|T^* \cap T |}{|T |}, R = \frac{|T^* \cap T |}{|T^* |}, F_1 = \frac{2PR}{P+R}, \end{aligned}$$

where *U* denotes the universal set of cells in our dataset, and $|\cdot |$ denotes the number of elements in the set.

The denoising autoencoder model and classifiers were implemented using Keras in Python. We conducted a grid search for the hyperparameters of the proposed denoising autoencoder; the number of epochs ($\varepsilon$): 100–300 with a step size of $+50$, learning rate ($\rho$): 0.0001–0.1 with a step size of $\times 10$, batch size: 3–18 with a step size of $+3$, feature vector (*Z*) size: 625–2500 with a step size of $\times 2$, and noise factor ($\sigma _n$): 0.1–0.5 with a step size of $+0.1$. The proposed model performed the best at: $\varepsilon$ of 200, $\rho$ of 0.01, batch size of 12, feature vector (*Z*) size of 1250, and $\sigma _n$ of 0.1. We also searched for hyperparameters of the STFT; the window size (*w*): 8–64 with a step size of $\times 2$, and the overlap size: 2–16 with a step size of $\times 2$. Based on a grid search, we determined the window size as 32 and the overlap size as 16. For the classifiers, we applied the max pooling layer with a pool size of 2 and (2, 2) in the 1D and 2D CNN, respectively, and the dropout rates were set to 0.2. The hyperparameters of the proposed classifiers were selected as follows; $\varepsilon$: 30–250 with a step size of $+20$, $\rho$: 0.0001–0.1 with a step size of $\times 10$, and batch size: 3–18 with a step size of $+3$. The CNN model had the best performance at: $\rho$ of 0.001, batch size of 15, and $\varepsilon$ of 50 for both the 1D and 2D CNN classifiers.

Furthermore, to demonstrate the necessity of neural network models, we compared the performance of the proposed classifiers with that of conventional ML algorithms, including support vector machine (SVM), logistic regression (logit), and multi-layer perceptrons (MLP). We searched for the optimal kernel of SVM among the linear, polynomial, and radial basis function (RBF) kernels, and the SVM classifier had the best performance with the linear kernel. We composed the MLP classifier with five hidden layers with 100 hidden units and the ReLU activation functions. The sigmoid activation was used in the output layer of this model, and its loss function was the binary cross-entropy loss. Parameters of the MLP model were initialized and updated using Xaiver normal initialization^52^ and Adam optimizer^44^, respectively, as with the proposed classifiers. We conducted the hyperparameter search for the MLP model in the same manner as the CNN classifiers. The MLP classifier performed the best at: $\rho$ of 0.001, batch size of 12, and $\varepsilon$ of 100.

### Effectiveness of the proposed system for automated cell classification

We investigated whether the proposed system can adequately and effectively distinguish cells from other micron-sized objects based on the following experiments: (1) classifying cell types with different diameters (e.g., RBCs and PNT1A cells), (2) distinguishing polystyrene microspheres with different diameters (e.g., 5 $\upmu$m and 10 $\upmu$m), and (3) classifying micron-sized objects with different physical properties and similar diameters (e.g., 5 $\upmu$m microspheres with RBCs and 10 $\upmu$m microspheres with PNT1A cells). Experimental results revealed the characteristics of cells that were reflected by the patterns of their backscattered signals.

#### Identification of red blood cells and cancer cells

We validated RQ 1 for the uniqueness of the backscattered signals of cells based on the effectiveness of the proposed models. We attempted to distinguish PNT1A cells from RBC using the proposed CNN classifiers. If backscattered signals reflect significant properties of cells, such as size and structural material, the classifiers will be highly accurate, and vice versa. Table 1 lists the experimental results.

As described in the upper part of Table 1, the time-frequency domain analysis exhibited the best performance among the three classifiers in terms of both the average accuracy and variance. Both the frequency domain and time-frequency domain analyses showed perfect accuracy on the second and third folds. Conversely, the frequency domain had a slightly lower accuracy than the other domains on the first fold. Compared with the other two classifiers, the proposed classifier for waveform signals had a slightly lower but similar accuracy on the first and second folds. However, the time domain analysis showed low performance on the third fold, even lower than that of conventional ML methods. We assumed that the testing data of the third fold may include signals that are only distinguishable using frequency domain features. In addition, the frequency domain features were more robust to unusual samples than the waveform. Nonetheless, on average, the proposed classifiers achieved a high classification accuracy ($\ge 0.88$), regardless of the data representations and accuracy metrics. Therefore, the experimental results indicate that the patterns of backscattered signals can be an effective feature for distinguishing particular types of cells (RQ 1).

Furthermore, we validated the necessity of deep learning-empowered models by comparing the proposed classifiers with conventional ML methods, as presented in the lower part of Table 1. We applied the same preprocessing methods, including denoising and augmentation, with the proposed classifiers to the comparison group for a fair comparison. The proposed classifiers outperformed the baseline methods, and their performance improvement was more vivid in the time- and frequency domain analyses than in the time-frequency domain. We assumed that combining the time- and frequency domain features may provide more abundant information for cells to the classifiers. Nevertheless, the distinct improvement indicates that the cell properties in backscattered signals are difficult to reveal using conventional methods, and the analysis capabilities of the CNN models are required. During the hyperparameter search, SVM with the linear kernel had a slightly higher accuracy ($\le 0.02$ in terms of $F_1$ measure) than with the other non-linear kernels. This point can be seen contrary to that the proposed CNN classifiers, which are non-linear models, significantly outperformed SVM with the linear kernel. However, considering the small performance gap between SVM kernels and differences in their model architectures, the higher model complexity of the CNN classifiers could make them more capable of expressing complicated correlations of signal features with cell characteristics than SVM. We can find a similar result in that SVM did not consistently outperform or underperform both logistic regression and MLP, which are non-linear. Therefore, we assume that (non-) linearity of the models was less influential to their effectiveness in analyzing backscattered signals than their architectures. MLP outperformed other baseline methods, such as SVM and logistic regression, for waveform and frequency spectra. However, MLP significantly underperformed for the spectrograms, demonstrating that MLP may not be suitable for analyzing sequential features, such as dynamic changes in the frequency spectra.

#### Identification of different-sized polystyrene micro-spheres

Next, the proposed model was used to classify two differently sized polystyrene microspheres (diameters of 5 and 10 $\upmu$m) to focus on the effect of size difference by excluding the possibility of structural material differences.

A previous experiment demonstrated that the proposed system could distinguish cells with different physical properties, including size and structural material. Next, we focused on the effect of size differences by excluding the possibility of structural material differences. We applied the proposed system to polystyrene microspheres of different sizes and the same structural material. The system classified two types of polystyrene microspheres based on their sizes (5 and 10 $\upmu$m) by analyzing their backscattered signal patterns. Table 2 presents the classification accuracy of the proposed models for polystyrene microspheres.

The proposed models exhibited high accuracy and low variance for classifying different-sized polystyrene microspheres. This result is consistent with our previous study^28^ and underpins that the diameters of micron-sized objects affect the patterns of backscattered signals. However, the results for polystyrene microspheres were also different from the experimental results for cells. In cell classification, frequency spectrum analysis outperformed waveform analysis, and temporal changes in the frequency spectra contributed to classification accuracy. However, the waveform and frequency spectrum analyses outperformed the spectrogram analysis in microsphere classification. This result indicates that the waveform signals and frequency spectra include sufficient features for determining the size differences in micron-sized objects. Moreover, we can suppose that features in the temporal changes in the frequency spectra are over-abundant for size classification. Basically, patterns of backscattered signals contain more diverse characteristics of cells than only cell diameters (e.g., structural material and properties of cell membranes), and spectrograms could represent these characteristics. This result indicates that spectrogram analysis is necessary to utilize backscattered patterns for more abstract automated diagnosis tasks.

#### Identification of similar-sized cells and polystyrene micro-spheres

We examined whether the proposed system can distinguish cells from similar-sized polystyrene microspheres to take a step further. As a previous experiment showed that backscattered signals reflect the size difference, this experiment investigated the effect of structural material differences by excluding the possibility of the size difference. We attempted to distinguish PNT1A cells ($10.10 \pm 0.88\;\upmu$m) from 10 $\upmu$m microspheres ($9.97 \pm 0.07\;\upmu$m) and RBC ($6.57 \pm 0.66\;\upmu$m) from 5 $\upmu$m microspheres ($4.98 \pm 0.06\;\upmu$m) using the proposed system. Table 3 presents the accuracy of the two classification tasks.

The proposed classifiers accurately distinguished RBC from 5 $\upmu$m microspheres while exhibiting high variance for PNT1A and 10 $\upmu$m microspheres, as summarized in Table 3. This result could be partially attributed to the fact that size differences between RBCs and 5 $\upmu$m microspheres were more significant than those between PNT1A cells and 10 $\upmu$m microspheres. Further, our dataset, which was relatively small-scaled, could be insufficient for the classifiers to understand the structural material differences between PNT1A cells and 10 $\upmu$m microspheres reflected by their backscattered signals. In future research, we will attempt to extend the range and scale of this dataset. However, despite the high variance, the proposed classifiers exhibited perfect accuracy in detecting PNT1A by two-fold among the three. Thus, we can assume that the classifiers can capture the structural material differences of micron-sized objects and size differences. Furthermore, in this experiment, the spectrogram analysis significantly outperformed the other cases, whereas both spectrum and spectrogram analyses exhibited flawless performance in the first experiment (Table 1). Temporal changes in the frequency spectra could represent more abundant information for the structural material of cells (and microspheres) than static frequency spectra or waveform signals.

### Efficacy of waveform, frequency spectrum, and spectrogram analysis

This section validates the efficacy of the frequency spectrum and its temporal changes for the cell characteristic analysis (RQ 2 and RQ 3). As listed in Table 1, both the frequency spectrum and spectrogram analyses could distinguish the two cell types from each other with high average accuracy and low variance. Basically, frequency domain features revealed the cell characteristics elicited by backscattered signals more effectively than time domain features. Additionally, Table 3 indicates that the time-frequency domain features were more useful than the other two data representations. These results were the same as those of the conventional ML methods (lower part of Table 1). However, we decreased the frequency resolution of the spectrogram analysis from 5000 to 513 by subsampling in the above experiments to addressed the time and space complexity. Simultaneously, we maintained the frequency resolution of the spectrum analysis at 5000. Therefore, the previous experimental results are inadequate for supporting RQ 3. To address this problem, we examined the accuracy of spectrum and spectrogram analyses based on a frequency resolution of 125–2000 with a step size of $+ 125$. Figure 5 shows the results of the experiment.

As shown in Fig. 5, the spectrogram analysis showed consistent and high accuracy regardless of the frequency resolution, and outperformed the frequency spectrum analysis. By contrast, the performance of the frequency spectrum analysis exhibited an unstable tendency. $F_1$ measure for the frequency spectrum analysis was lower than 0.70 until the frequency resolution became 1750. Particularly , its precision was far more severe than its recall. When the resolution of the frequency spectra was lower than 2000, the cell characteristics reflected by the backscattered signals vanished. In this case, the previous experimental results in Tables 1, 2 and 3 indicate that the temporal analysis of frequency spectra discovered distinct aspects of cell properties, which are different from the static analysis. Furthermore, reducing frequency resolution makes temporal analysis computationally more efficient than static analysis.

### Effectiveness of denoising autoencoder and Gaussian noise injection

We conducted an ablation test for the noise reduction methods used in the proposed system. The remarkable performance of the proposed system can cause the misunderstanding that the proposed CNN models are overexerted, and the conventional features might be capable of cell classification. However, the backscattered signals of cells are extremely noisy, and extracting essential features under noise with conventional heuristics is more difficult . We attempted to demonstrate this through an ablation test for the denoising autoencoder and Gaussian noise injection. We compared the performance of the proposed system employing the proposed denoising methods (DN+) for classifying RBCs and PNT1A cells with a case using only the denoising autoencoder (DN) and another case without any denoising technique (Raw). Table 4 presents the experimental results of the contributions of the proposed denoising methods to the accuracy of our automated cell-type classification system.

As listed in Table 4, the three cases exhibited similar accuracies in the waveform and spectrogram analyses. However, DN+ distinctly outperformed the other two cases in the frequency spectrum analysis. This result indicates that our noise reduction methods effectively recover the frequency domain features of the backscattered signals from the cells. Nevertheless, the proposed denoising autoencoder could not contribute to the classification accuracy without Gaussian noise injection. Data augmentation using Gaussian noise injection enabled training the proposed autoencoder and classifiers to distinguish significant signal features from noise. Furthermore, all three classifiers were trained using the same data augmentation method (not only in the denoising step), but noise only affected the frequency spectrum analysis. Thus, we can conjecture that the temporal features of backscattered signals are more robust to noise than the frequency domain features.

## Discussion and concluding remarks

This study aimed to automatically classify live cells based on cell types by analyzing the patterns of the backscattered signals of the cells. A previous study^28^ applied the label-free acoustic sensing technique to determine the size differences between RBCs and PNT1A cancer cells by measuring the IB coefficients of the backscattered signals with manual postprocessing. However, automatically analyzing the patterns of the backscattered signals enabled us to avoid time-consuming processes and possible errors caused by manual analysis. This study demonstrated a novel automated cell-type classification system by combining label-free acoustic sensing of a trapped single object^28^ with a 1D convolutional autoencoder and CNN classifiers. The experiments and research questions were designed to validate the effectiveness of each module of the proposed system. First, we verified the effectiveness of the patterns of backscattered signals for classifying cell types by applying the proposed system to two types of cells (RBC and PNT1A) and two types of polystyrene microspheres (5 and 10 $\upmu$m). Using cells and microspheres, we conducted three experiments to identify: (1) RBCs and cancer cells, (2) polystyrene microspheres of different sizes, and (3) similar-sized cells and polystyrene microspheres. We also compared the three preprocessing methods to examine whether the types of features in backscattered signals (e.g., time and frequency domains) were correlated with the physical properties of micron-sized objects. The experimental results indicated that the backscattered signal patterns reflect cell diameters and other physical properties, such as structural material differences, which reveals the importance of understanding their relation with fundamental molecular, architectural, and behavioral changes associated with cell state and disease processes^63,64^. Consequently, both time- and frequency domain features were significant for analyzing cell characteristics. In terms of ML models, the necessity of the CNN classifiers was demonstrated by comparing their performance with conventional ML models, and the efficacy of the denoising autoencoder was validated based on ablation tests.

In Tables 1 and 2, the size difference could be one of the significant factors affecting the patterns of the backscattered signals from particles or cells. Consequently, we arrived at a conclusion that is consistent with a prior study^28^. However, as summarized in Table 3, the proposed system exhibited high accuracy in distinguishing cells from similar-sized polystyrene microspheres. This result indicates that the physical properties of the structural material also affect the backscattered signal patterns, not only the sizes. The proposed system accurately distinguished RBCs from 5 $\upmu$m microspheres, despite the high variance in classifying PNT1A and 10 $\upmu$m microspheres. This result might be due to size differences as well as the structural material. Although the size difference between RBCs and 5 $\upmu$m microspheres was slightly greater than the difference between PNT1A cells and 10 $\upmu$m microspheres, difference of microstructure along with cell mechanics may be contributed as a secondary factor. Prior studies have shown that cell nucleus is the stiffest with a densely packed object compared to the surrounding cytoplasm^65,66^ and the average diameter of the nucleus is approximately from 5 to 20 $\upmu$m in mammalian cells^67^, which is similar to or even greater than the average diameter of RBCs. In contrast, when mature, RBCs in mammals do not have cell nucleus^68^ and they are easily deformed to efficiently travel through capillaries^69^. Furthermore, in optics, cell nuclei have different refractive index and mass density compared to the cytoplasm^70,71^. Therefore, such differences of microstructure along with cell mechanics may be involved in the high variance for classifying PNT1A cells and 10 $\upmu$m microspheres compared to RBCs and 5 $\upmu$m microspheres, but this will be intensively explored with large-scale datasets in the future.

The proposed system exhibited excellent accuracy for cell classification, whereas the conventional methods showed low accuracy, as listed in Table 1. These results indicate that the use of deep learning-empowered models can be overexerted, and a well-designed system with conventional methods can achieve a comparable performance. However, subsequent experiments (Fig. 5 and Table 4) indicated that the proposed system (particularly the spectrogram analysis case) can overcome low frequency resolution and the absence of denoising. Because the backscattered signals from cells were tiny with unwanted noise, it is difficult to expect that conventional ML algorithms and heuristic-based manual methods can discover essential features related to cell characteristics among noises and insignificant reflections. In addition, these results indicate that combining features in the time and frequency domains improves the accuracy of discovering cell properties, robustness to noise, and resolution reduction. Basically, temporal changes in frequency spectra might contain more abundant and distinctive information on cell physical properties, including size and structure material, than static frequency spectra or waveform signals.

The proposed system can classify live cells accurately without using manual postprocessing, compared with the previous study^28^. Nevertheless, the proposed system has a few limitations that should be addressed in future research. First, our experiments were conducted using two types of cells (RBC and PNT1A) and two types of microspheres (5 and 10 $\upmu$m). Although we demonstrated that our system could consider various cell characteristics (not only diameters) by classifying cells and microspheres with similar sizes, further research should examine more cell types with various sizes to validate this notion. The number of cells and backscattered signals should also be increased. In this study, we collected 154 signals from 24 cells and 146 signals from 31 polystyrene microspheres. Although the cross-validation results showed that the high accuracy of the proposed system did not result from overfitting, the number of cells and samples was not sufficient to show the diversity of live cells. By extending the dataset, our future research will focus on discovering correlations between physical and functional cell characteristics, and patterns of backscattered signals from cells. Discovered correlations can be elucidated and subsequently translated into clinical medicine involving disease progression and treatment response.

## Data Availability

The datasets used and/or analyzed during the current study are available from the corresponding author on reasonable request.
